# The influence of dexamethasone on postoperative nausea and vomiting in patients undergoing gynecologic laparoscopic surgeries: A randomised, controlled, double blind trial

**DOI:** 10.4274/tjod.13471

**Published:** 2014-12-15

**Authors:** Sara Asadollah, Mansoureh Vahdat, Payman Yazdkhasti, Nasrin Nikravan

**Affiliations:** 1 Tehran University of Medical Sciences Rasool-E-Akram Hospital, Clinic of Obstetrics and Gynecology, Tehran, Iran; 2 Tehran University of Medical Sciences Rasool-E-Akram Hospital, Clinic of Anesthesiology and Pain, Tehran, Iran; 3 Tehran University of Medical Sciences, Medical Student Research Committee, Tehran, Iran

**Keywords:** dexamethasone, postoperative nausea and vomiting, gynecologic laparoscopic surgery

## Abstract

**Objective:**

Dexamethasone, as a part of multimodal approach, can decrease nausea and vomiting following laparoscopy in high risk patients. We performed this study to find out whether the dexamethasone can improve postoperative nausea and vomiting (PONV) in patients undergoing gynecology laparoscopic surgeries.

**Materials and Methods:**

In this double-blind randomized clinical trial, 91 patients who underwent gynecologic laparoscopic surgery in Rasool Akram hospital in Tehran during 2011-2014 were enrolled. Fourty-four patients received 8 mg dexamethasone (study group) and 47 patients received 10 mg metochlopramide (control group) intravenously after intubation. Outcome parameters including age, weight, height, cause of hospitalization, drugs, Last Menstrual Period (LMP), Blood Pressure (BP), Heart Rate (HR), Respiratory Rate (RR) and oxygen saturation, Visual Analogue Scale (VAS) score, nausea and vomiting were entered to SPSS (v.16) and were analyzed.

**Results:**

Eighyt-eight American Society of Anesthesiology (ASA) class 1-2 patients between 25-39 years old were analyzed. There was no difference in vital signs during and post operation (BP, HR, RR and O_2_ saturation) between these two groups (p value>0.05).

There was no significant difference between VAS score at 4 and 24 hours after the operation (14% vs. 17.8% and 7% vs. 6.7%, respectively, p>0.05). Incidence of PONV in 4 hours was significantly lower in dexamethasone group (11.6% vs. 55.6% p<0.0001), while there was no significant difference in 24 hours (23.3% vs. 22.2%, p>0.05) and also need to anti-emetic drugs wasn’t significantly lower in study group (p>0.05).

**Conclusion:**

We conclude that dexamethasone can relieve PONV after gynecologic laparoscopic surgery.

## INTRODUCTION

Nowadays, laparoscopy is one of the most common surgery methods which is used for diagnosis and treatment all over the world. Laparoscopic surgery has various indications in almost all surgeries. It has many advantages such as faster recovery and shorter hospital stay^(1,2,3)^.

Despite significant advances in anesthesiology, postoperative nausea and vomiting (PONV) occurs mainly in the first day after operation^(4)^ and cause prolonged postanesthesia care unit (PACU) stay which increases medical costs^(5)^. According to available reports, 20-30% of patients experience nausea and vomiting after various types of surgeries^(6,7,8,9)^. This incidence is about 70% in high-risk patients^(10)^. PONV is also one of the most common reasons for poor satisfaction in patients undergoing this surgery^(11)^.

Glucocorticoids have analgesic, anti-inflammatory, immunomodulatory, and also antiemetic effects^(12)^. Many randomized controlled clinical trials have been examined the effects of administration of single dose glucocorticoid on PONV^(13,14,15)^. Recent studies showed that use of dexamethasone, as a part of multimodal approach, can decrease nausea and vomiting in high risk patients^(16,17,18)^. In spite of these results, some studies reported controversial conclusions^(19,20)^. However, there is still no consensus on whether it should be used routinely for prophylaxis of PONV.

We therefore performed present study to investigate whether the dexamethasone can improve PONV in patients undergoing laparoscopic gynecologic surgeries.

## MATERIALS AND METHODS

In this double-blind randomized clinical trial, 91 patients who underwent gynecologic laparoscopic surgery such as tubal ligation, myomectomy or ovarian cystectomy in Rasool Akram hospital of Tehran, Iran, during December 2012 to January 2014 were recruited. The Institutional Ethics Committee approved this study, and informed written consent was obtained. Inclusion criteria were: Women classified as American Society of Anesthesiology (ASA) physical status 1-2, lack of allergy to metoclopramide, not use anti-depressants and anti-emetics in 24 hour before surgery.

Exclusion criteria included: Pregnant or lactating women, weight over 90 kg, conditions such as malignant hypertension, hepatic dysfunction, pheochromocytoma and seizure, symptoms of extrapyramidal, mechanical ileus, need post operation ventilator or NG tube.

A total of 91 Patients were randomized to receive either 8 mg dexamethasone intravenously (study group) or 10 mg metoclopramide intravenously (control group) after intubation. Patients and Anesthesiologist were blinded to the study groups. Fourty-four patients were randomized to the study group and 47 patients to the control group. One patient from study group and 2 patients from control group who needed laparotomy due to adhesions during surgery, were excluded (chart 1). Operative and anesthetic techniques and received drugs were similar in two groups. All patient received crystalloid 5 cc/kg before anesthesia. Patients were monitored by anesthesiologist in the operation room and baseline blood pressure (BP), heart rate (HR), respiratory rate (RR) and pulse oximetry were measured. Midazolam 0.02 mg/kg as premedication, fentanyl 2 mg/kg, propofol 2 mg/kg and atracorium 0.5 mg/kg were used for anesthesia in all patients. Propofol 150 mg/kg/min and fentanyl 0.4 µg/kg/min were used for maintenance. Atracorium 15 mg was repeated every 15 minutes for muscle relaxation.

Laparoscopic surgeries were performed with video guidance using 3 trocars. Pressure of CO_2_ into abdomen was 10-14 mm Hg. Neostigmine 0.04 mg/kg and atropine 0.02 mg/kg were prescribed to reverse patients at the end of operation. Patients were extubated after operation.

Patients were transferred to recovery room after operation and their BP, HR, RR and O_2_ saturation were checked. They transferred to ward after stabilization.

Outcome parameters including age, weight, height, cause of hospitalization, drugs, last menstruation period (LMP) were entered to checklists from patient’s documents.

Severity of pain after operation was recorded using visual analogue scale (VAS score 0-10). Nausea was defined a subjective unpleasant sensation associated with the awareness of the urge to vomit. Vomiting was the forceful expulsion of gastric contents from the mouth. Nausea and vomiting were evaluated on a two-point verbal scale (0, none; 1, nausea or vomiting). Incidence of PONV and the intensity of postoperative pain were recorded after 4 and 24 hours. Anti-emetics (such as metoclopramide 10mg/IM/PRN or promethazine 25 mg/IM/PRN) was prescribed if nausea and vomiting continued. Any patient complaining of pain or Visual analog scale (VAS>4), 100 mg of suppository of diclofenac was received and if needed, the same dose was repeated till the patient was free of pain.

### Statistical Analysis

The primary outcome variable of this study was the incidence of PONV. Previous studies^(6,8)^ reported an incidence of PONV of 26% and a reduction of this incidence by about 50% with dexamethasone. Based on these data, this study had to include at least 44 subjects in each group to have a power of 0.8 and an α of 0.05.

Data were cleaned, coded and entered in SPSS version 16. The distribution of continuous variables was examined by Kolmogorov-Smirnov test. Presence of statistical association between dependent and independent variables was assessed using Chi-square tests and student t-test was used to compare the means of continuous variables. For pain score comparisons Mann-Whitney U-test were used. Data were expressed as mean values (Standard deviation) or median (Inter quartile range) or number (percentage). P-values of <0.05 were considered statistically significant.

## RESULTS

**Figure 1:**
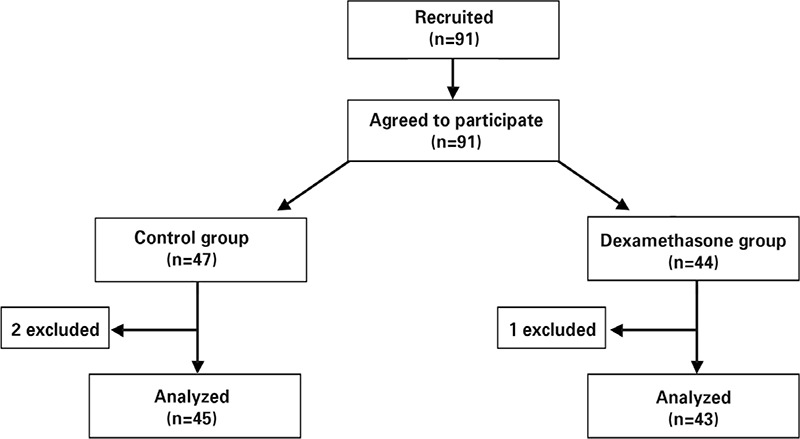
Flow diagram of enrollment, design and analysis of study participants Control group received metoclopramide

From 91 patients who entered the study, 3 individuals underwent laparotomy due to adhesions; so 88 patients were analyzed (Figure 1). Mean age of these patients was 31.7 (±3.8) years. There was no significant difference in age, Weight, BMI, operation duration, type of surgery and duration of anesthesia between two groups of our study (p value>0.05) (Table 1, 2). There was not any significant difference also in vital signs (BP, HR, RR and O_2_ saturation) between these two groups (p value>0.05).

As it was shown in Table 3, VAS score in 4 h and 24 h after the operation was statistically lower in dexamethasone group (p<0.05).

Twenty-five patients (55.6%) in control group, had PONV while 5 (11.6%) in study group during 0-4 hours but during 4-24 hours post operation, 10 (23.3%) in dexamethasone group and 10 (22.2%) in control group experienced PONV. Frequency of nausea and vomiting in 4 hours was significantly different between two groups of study (p value<0.0001), while there was no significant difference in 24 hours (p value> 0.05).

Anti-emetics (promethazine 25 mg/IM or metoclopramide 10 mg/IM) was prescribed in case of nausea and vomiting after operation. Analysis shown that need to anti-emetics in study group wasn’t significantly lower than control group (9.3% vs. 22.2% p=0.098), (Table 3).

There was not seen any side effect of these medications in our patients.

## DISCUSSION

Nowadays, prevention and treatment of postoperative pain and operation complications such as nausea and vomiting following laparoscopy are important concerns. Non-opioid analgesics are usually used to reducing postoperative pain^(21)^. Although PONV is not a life threatening problem, it may increase hospital stay and medical costs^(5)^ so reduction of this complication is very important.

At least four major receptor systems were reported that are involved in etiology of nausea and vomiting, including cholinergic or muscarinic, dopaminergic (D2), histaminergic (H1) or serotonergic (5-HT3) receptors^(22)^. 5HT-3 antagonists (eg. granisetron, ondansetron), anticholinergics (eg. Scopolamine) and dopamine antagonists (eg. Metoclopramide) are effective antiemetics but clinical use of these drugs due to their costs and side effects was limited^(23,24)^.

At the first time, dexamethasone has been shown to be effective in patients undergoing cancer chemotherapy in 1981^(25)^. Previous published studies showed that dexamethasone alone or combination with other drugs (such as Granisetron, Ondansetron) can decrease nausea and vomiting in high risk patients^(16,17,18)^.

This study basically demonstrated that administration of 8 mg dexamethasone before operation significantly reduced PONV in 4h. The type of surgery and anesthesia protocol were similar among the groups, so the difference in incidence of PONV between these groups was directly related to the administration of dexamethasone. This result is in accordance with some authors^(26,27)^, so dexamethasone as a corticosteroid can be used in the prevention and treatment of nausea and vomiting after the operation.

But this study didn’t show any difference of PONV incidence in 24 h so delayed emesis (up to 24 h) cannot be controlled by dexamethasone. This finding is different from other studies showed that dexamethasone can reduce delayed emesis more than other antiemetics^(28,29)^. Maybe due to use of combination therapy of dexamethasone with other antiemetics in these studies that is more effective than single drug alone. Another possible reason is that the effect of dexamethasone can be reduced over the time.

Although the anti-inflammatory and antiemetic effects of dexamethasone are well known, their effects on postoperative pain remain unclear^(30,31)^.

We found that pain score in the first 4 h and 24 h of the postoperative period was lower in dexamethasone group, compared with control group. But incidence of VAS score≥4 wasn’t significantly different between two groups at 4 and 24 h.

Results of a systematic review about effects of perioperative single-dose glucocorticoid in several minor and major surgical procedures showed that administration of glucocorticoid has no or limited analgesic effects in major abdominal surgery, but may have significant effects in minor procedures^(13)^.

In present study, we found no side effects following use of dexamethasone in prevention and treatment of PONV. Similar to our study there are no reports of dexamethasone related complications in any type of surgery for management of PONV^(13,22)^. Therefore, evidence showed that single preoperative dose of dexamethasone is safe and has no significant side effects and can be used without any concern in the management of PONV.

According to reducing pain, nausea and vomiting in the first hours after the operation, patients who received dexamethasone can be discharged earlier so treatment costs and complications could be decreased.

There are some limitations in our study, mostly due to small sample size and lost to follow-up more than 24 h.

In summary, we conclude that dexamethasone can relieve post¬operative nausea and vomiting. Since this regimen is safe and free of apparent side effects, we suggest that preoperative dexamethasone could be used as routine in otherwise healthy patients undergoing elective laparoscopic surgeries.

## Figures and Tables

**Table 1:**
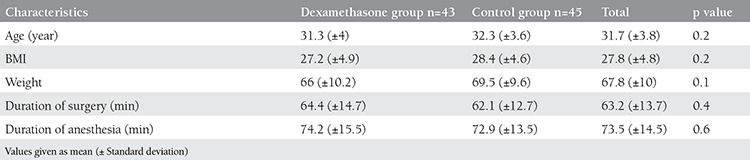
Demographic characteristics of the Patients

**Table 2:**
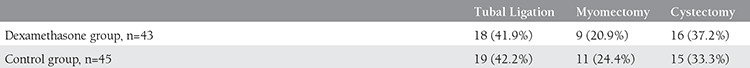
Types of gynecologic laparoscopic surgery

**Table 3:**
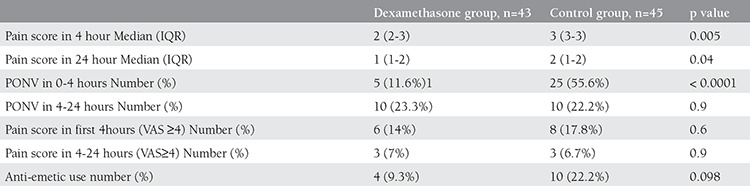
Nausea and Vomiting, pain score and ant-emetic use after laparoscopy
